# Deep learning for prediction of population health costs

**DOI:** 10.1186/s12911-021-01743-z

**Published:** 2022-02-03

**Authors:** Philipp Drewe-Boss, Dirk Enders, Jochen Walker, Uwe Ohler

**Affiliations:** 1 Berlin Institute for Medical Systems Biology, Max Delbrück Center for Molecular Medicine in the Helmholtz Association, Robert-Rössle-Strasse 10, 13125 Berlin, Germany; 2 Institute for Applied Health Research (InGef), Spittelmarkt 12, 10117 Berlin, Germany

**Keywords:** Deep learning, Health insurance claim records, Health cost prediction

## Abstract

**Background:**

Accurate prediction of healthcare costs is important for optimally managing health costs. However, methods leveraging the medical richness from data such as health insurance claims or electronic health records are missing.

**Methods:**

Here, we developed a deep neural network to predict future cost from health insurance claims records. We applied the deep network and a ridge regression model to a sample of 1.4 million German insurants to predict total one-year health care costs. Both methods were compared to existing models with various performance measures and were also used to predict patients with a change in costs and to identify relevant codes for this prediction.

**Results:**

We showed that the neural network outperformed the ridge regression as well as all considered models for cost prediction. Further, the neural network was superior to ridge regression in predicting patients with cost change and identified more specific codes.

**Conclusion:**

In summary, we showed that our deep neural network can leverage the full complexity of the patient records and outperforms standard approaches. We suggest that the better performance is due to the ability to incorporate complex interactions in the model and that the model might also be used for predicting other health phenotypes.

**Supplementary Information:**

The online version contains supplementary material available at 10.1186/s12911-021-01743-z.

## Background

Health care expenditures are one of the biggest expenses in Germany and optimally managing these cost has great economical importance. Therefore, methods for accurate patient-level prediction of future health care cost are needed to provide the basis for decision making. As medical costs reflect the development of health over time, and health in turn is influenced by many factors such as social demographics, previous medical history, environmental influences, genetics but also by random events such as accidents, predicting the future health is inherently challenging. Consequently, accurately predicting health cost is a challenging problem. Existing work on prediction of health cost can be divided into two categories [1]: (1) Rule based prediction methods, in which decision rules of an algorithm to predict future costs are manually defined. The disadvantage of this approach is that it requires deep domain knowledge and that the capability of resulting models to reflect complex relations in the data is limited. (2) Supervised learning based methods (e.g. linear regression models, random forests or support vector methods) that learn to predict future cost from the data [1–6]. These methods have the advantage that they are not limited in their expressiveness as rule based methods are. However, they typically require large datasets for training. For training of these methods, health insurance claims records are an appealing data source. They cover most of the health care expenditures of the patients and have the advantage of having sample sizes that allow fitting rich models. Additionally, they contain detailed information on patients, such as the medical history and social demographic information. The challenges of this data is that it is high dimensional, that there are many hidden interactions between variables, and that the data is often not normally distributed [7]. The aforementioned supervised learning methods are believed to typically not leverage the potential of population scale data to detect complex patterns [8]. Recent developments in deep learning techniques, such as novel deep neural network architectures and numerical approaches to fit the networks, promise to address some of these challenges. Deep learning has been successfully applied in the medical domain to task such as dermatologist level detection of skin cancer [9], prediction of various clinical outcomes from electronics health records [10], or the detection of diabetic retinopathy from retinal fundus photographs [11], showing the potential of this technology.

**Fig. 1:**
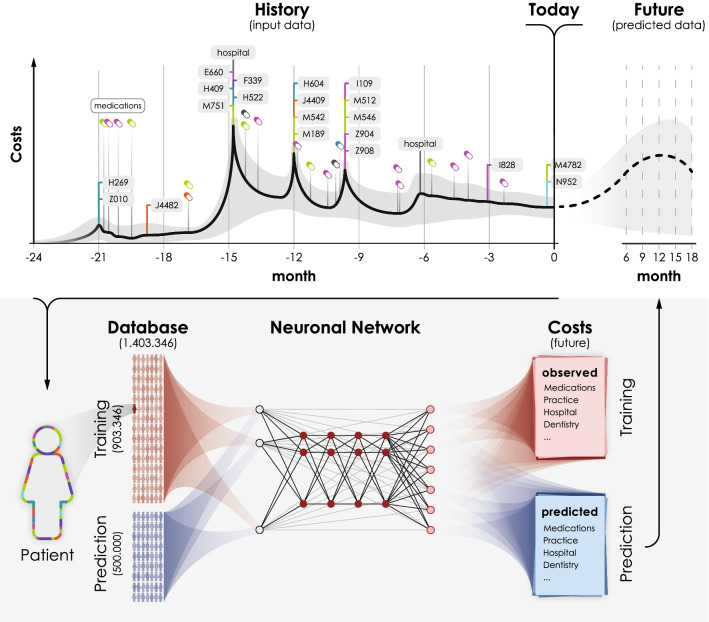
Schematic diagram of workflow. A neural network is trained to predict from health insurance claim data (input data) of a subset of the population (shown in red) the future costs. The neural network can then be used to predict the cost of a different subset (shown in blue) of the population based on their health insurance claim data

We present a novel deep neural network architecture to predict future health care cost from health insurance claims records (See Fig. 1). This network architecture allows to fully capture the richness of the medical data in health insurance claims and can be fitted on a standard workstation with 64GB of RAM. We compare it on health insurance claim records of $\sim 1.4$ million patients from German statutory health insurances against various standard methods and show that it outperforms existing approaches. It also is better identifying patients at risk than standard linear regression approaches and the Morbi-RSA approach used by the German Federal Office for Social Security. Finally, we show how the parameters of the network can be interpreted and that the network uses medically relevant features for its prediction.

## Methods

### Data

This study is based on data of the Institute for Applied Health Research Berlin (InGef) database, which contains anonymised longitudinal claims data of more than 60 German statutory health insurances. Claims data of the years 2010 to 2017 for a sample of about $1'403'346$ insurants was used, which is representative for the German population with respect to age, sex and state of residence. Besides sociodemographic information, the database contains information on hospital stays, outpatient physician visits, drug prescriptions and remedies and aids including costs in each of the sectors. Further details of the database can be found elsewhere [12]. An approval of an ethics committee or informed consent of the patients was not required for the conduct of this study since all patient- and provider-level information are anonymised to comply with German data protection regulations and German federal law. In the remainder of this manuscript, we will refer to the period ranging from Q1 2010 to Q4 2015 as the observation period and to the period from Q3 2016 to Q2 2017 as the evaluation period.

### Data representations

The input for the machine learning algorithms was formatted in the following manner for each patient in a given quarter: Each numerical value was kept as a feature. Dates were coded per quarters since Q1 of 2010. Categorical values, such as International Statistical Classification Of Diseases And Related Health Problems, 10th revision, German Modification (ICD-10-GM) codes, Anatomical Therapeutic Chemical (ATC) codes, Diagnosis Related Group (DRG) codes, German procedure classification (OPS), physician subject group key (FG) and schedule of fees for physician outpatient services (GOP) codes or sex, were coded using a one-hot encoding (i.e. if *n* possible categories $k_1,\ldots ,k_n$ were possible, the observation of category $k_j$ was coded by a *n*-dimensional vector that was 1 at the index *j* and 0 everywhere else). If multiple codes for a one-hot-encoded category (e.g. ICD-10-GM or ATC) were observed in a quarter, the representing vectors were added. We then concatenated all vectors and features to obtain one 91’470 dimensional vector per quarter and per patient. Finally, the resulting vectors for all quarter (n = 24) were concatenated into a single vector of dimension 24*91’470 =2’491’470 representing a patient in the observation period. In order to accelerate model fitting, we only considered variables that had more than $1'000$ entries over all patients in the observation period. This lead to a vector of dimension 24*13’876 = 333’024 for representing each patient in the observation period.

### Model definition

**Fig. 2:**
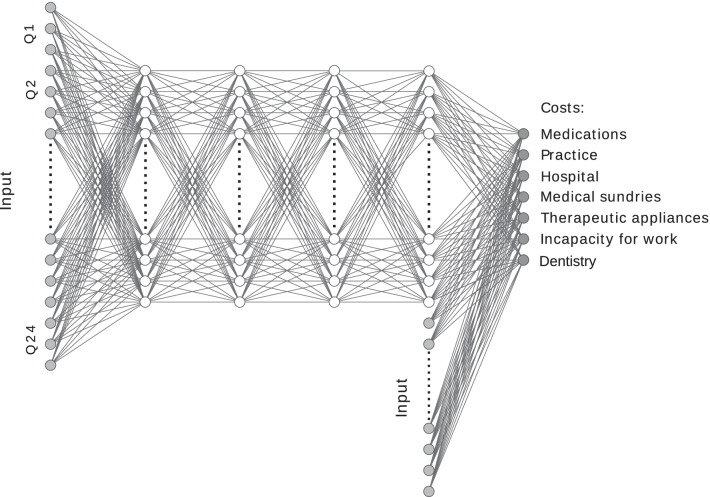
Network architecture: Shown is the architecture of the proposed deep neural network. Shown in (light grey) are the input features. Shown in (dark grey) are the target variables of the network. The (white) nodes are the internal nodes of the network

We used a multilayer perceptron deep learning model with four hidden layers (See Fig. 2). Our analysis (See Table 1) suggests that this is the optimal depth according to the mean absolute prediction error (MAPE). The first four layers had each 50 neurons. In the last hidden layer the original input was concatenated to the hidden vector and fed to the last layer, which had seven neurons to predict seven cost categories (Medications, practice, hospital, medical sundries, therapeutic appliances, compensation for incapacity to work and dentistry). Intuitively, concatenating the original input to the last hidden layer allows the network to model simple relationships between the input and output using a multivariate regression and the residuals using a complex deep learning model. All layers used the ReLU-activation function [13] and a dropout [14] rate of 0.25 during training (See Supplemental Material for the code for training).

**Table 1:** Performance assessment depending on the network depth

	r	$\rho$	MAE	$r^2$	CPM
Neural network (depth 2)	0.518	0.566	2170.83	0.265	0.27-
Neural network (depth 3)	0.525	0.622	2065.49	0.271	0.304981
Neural network (depth 4)	0.524	0.631	2013.35	0.264	0.323
Neural network (depth 5)	0.530	0.622	2066.74	0.270	0.304561
Neural network (depth 6)	0.526	0.617	2165.04	0.275031	0.271482
Neural network (depth 7)	0.525	0.616	2165.54	0.272261	0.271315
Neural network (depth 8)	0.497	0.601	2414.74	0.244568	0.187461

Evaluation of methods using: Pearson’s correlation (r), Spearman’s correlation ($\rho$), mean absolute error (MAE), R squared ($r^2$) and Cumming’ s Prediction Measure (CPM)

We compared the deep learning model to three baseline models. (1) The average cost per year in the previous 6 years as prediction for the cost in the evaluation period. (2) The costs in the last year of the observation time as prediction for the cost in the evaluation period. (3) A two-stage approach where first, a multivariate ridge regression with regularisation parameter $\lambda =0.1$ was trained to predict the seven different cost types in the evaluation period. Second, the seven predicted cost types are summed to compute the total sum in the evaluation period for each patient. For model assessment, we predicted separately the seven (see above) different cost domains using a the proposed neural network. We then summed all predicted costs except the cost to compensate for incapacity to work for model assessment, in order to make the cost comparable to costs reported for the Morbi-RSA [4, 15]. Furthermore, we performed model ensembling for the ridge regression and the neural network (i.e. the identical model was trained five times on the same data with different random seed parameters and the average of the predictions of all five models was computed to obtain the final prediction).

### Model fitting

For model fitting we used the first $903'346$ patients (training set). During model fitting, we minimized the *l*2-loss between the future and the predicted costs using ADAM [16], which is an extension of stochastic gradient descent. For ADAM we have used a learning rate of 0.001 and gradient normalization with parameter 1.0. Both the ridge regression and the deep learning model were trained for 25 epochs. For training of the ridge regression a batch size of 128 was used and for training of the neural network a batch size of 32 was used.

### Implementation

All models have been implemented in python and keras [17].

### Evaluation criteria

We evaluated the ability of the models to predict from the observation period of a set of patients that were not used for training the model (evaluation set) the summed cost per patient in the evaluation period. To assess the model quality, we used the following quality criteria: Pearson’s correlation coefficient, Spearman’s correlation coefficient, the mean absolute error and Cumming’s Prediction Measure (CPM). The performance was evaluated on the subset of $357'239$ of the $500'000$ held out patients (test set) that where alive in the observation period and either died or were still insured on at least one day in the evaluation periods.

We further assessed how well the methods could be used to identify patients with changing costs. As this is indicating a change of health status or treatment, these patients could benefit from preventive interventions. To this end, we divided our test set patients into three groups. Those for which the cost decreased more than 100-fold between the last year of the observation period and the first year of the prediction period; those for which the cost increased more than 100-fold; and the remaining patients. In order to not include patients with overall low cost in the two group (e.g to not include patients that change from 0.01 to 10.0 Euro) that have strong cost changes, we added 10 Euros to the overall cost before computing the fold change. We then computed the area under the precision-recall curve (auPRC) for identification of patients with increasing, resp. decreasing costs from all patients. To understand for which cost range the respective methods performed best, we computed the error of the prediction in dependence of the cost.

### Sensitivity analyses

We investigated how the performance of the neural network depends on the amount of available training data. To this end, we trained the model on only $100'000$, $200'000$, $300'000$, $400'000$, $500'000$, $600'000$, $700'000$, $800'000$ and $900'000$ patients. Furthermore, we investigated how the length of the observation time affects the predictive performance. Therefore, we trained the model also for each of the patient sets using the data from one to six years up to the end of the observation period.

### Feature identification

An important application of predictive models is to identify relevant features in the data and to understand their effect on the prediction. This allows for example to identify and quantify risk factors. A common approach in linear models is to identify the weights that have a large absolute value as they correspond to the features that have as strong impact on the prediction. For deep neural networks it has been shown that this strategy is suboptimal [18] as it does not capture the interactions between features that the neural network uses. Here, we therefore used a strategy called integrated gradients [18] that is more robust. We determined the average integrated gradients of all patients in the evaluation set. Furthermore, we divided the mean integrated gradient by the number that the actual feature was nonzero, to account for the fact that not all features are equally abundant. We did not show codes in the results that allow identification of health insurance companies which contributed to the study database.

## Results

To establish a baseline, we first compared the performance of all methods to predict costs. We found that the neural network was able to better predict future costs than ridge regression or the other two standard models in all considered measures as shown in Table 2. Furthermore, we found that ensembling several training runs provides an additional small improvement.

**Table 2:** Performance assessment

	r	$\rho$	MAPE	$r^2$	CPM
Spendings in last year	0.418	0.551	2403.30	$-$0.005	0.191
Mean of previous spendings	0.464	0.547	2078.76	0.200	0.301
Ridge regression	0.514	0.610	2126.03	0.260	0.285
Neural network	0.524	0.631	2013.35	0.264	0.323
Ridge regression (ensemble)	0.517	0.611	2116.67	0.265	0.288
Neural network (ensemble)	**0.527**	**0.632**	**2004.33**	**0**.**266**	**0.326**
Morbi-RSA model (2018)$^*$	na	na	2267.60	0.258	0.242
Morbi-RSA full model$^*$	na	na	2233.53	0.263	0.253

The best performance for each evaluation criterion is shown in bold

Evaluation of methods using: Pearson’s correlation (r), Spearman’s correlation ($\rho$), mean absolute prediction error (MAPE), R squared ($r^2$) and Cumming’s Prediction Measure (CPM). Performance for the Morbi-RSA models on a different data set ($^*$) where obtained from [4, 15]. Correlation values where not available (na) for these models

**Fig. 3:**
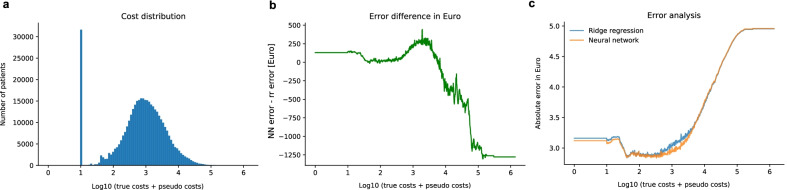
Error analysis: Shown is the histogram of total cost (**a**), the log10 absolute error based on the true cost of the ridge regression and the neural network (**b**) as well as the difference between the neural network error and the ridge regression error (**c**)

To better understand in which cost regimes the neural network and the ridge regression performed better, we studied the average absolute error in Euros depending on the true costs. The neural network performed better for patients with total costs lower than $\sim 10'000$ Euro, whereas the ridge regression performed better for patients who were more expensive (See Fig. 3a, b).

**Fig. 4:**
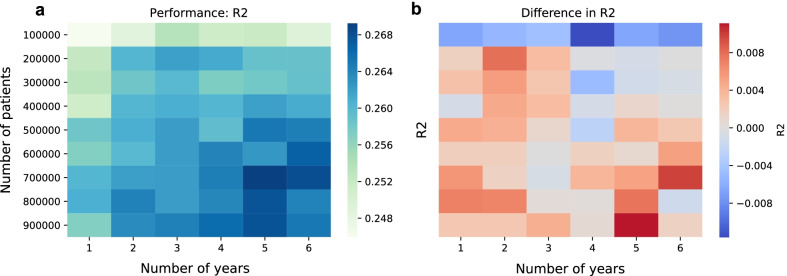
Dependence of performance on patient number and observation time: Shown is the performance ($r^2$) of the neural network depending on the patient number and the length of the observation period in years (**a**). Shown in (**b**) is the difference between the *r*2 of the neural network and of the ridge regression

As sensitivity analyses, we studied how the number of samples in the training set and the length of the observation period affect the performance of the prediction for the neural network. Our analyses showed that as the number of patients increased the predictive performance, as measured by $R^2$, increased. The same was true when the observation time increased (See Fig. 4a). A similar picture can also be seen for the Spearman and Pearson correlation (See Additional file 1: Fig. S1). We compared this to the performance of the ridge regression (See Fig. 4b). We found that at $100'000$ patients the $r^2$ of the neural network was lower than for the ridge regression but that for larger sample sizes the neural network had in general a higher $r^2$.

**Fig. 5:**
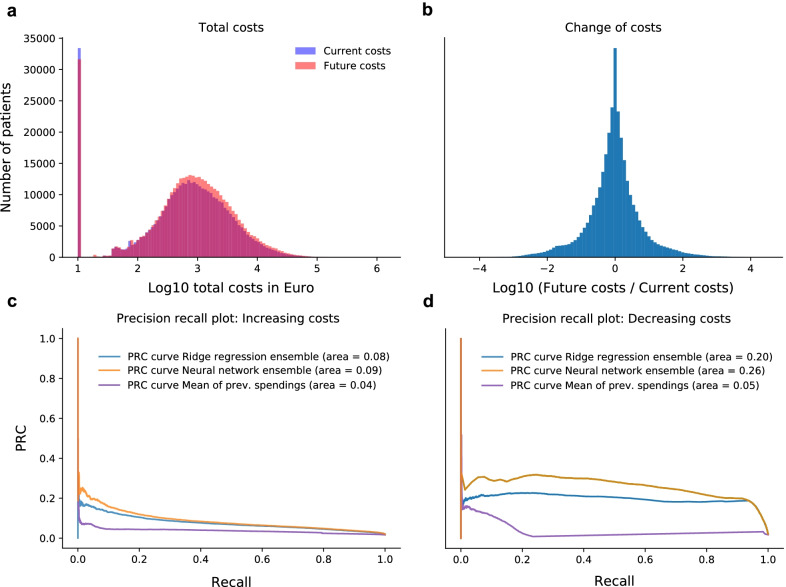
Cost change prediction: Shown are the raw cost (**a**) in the last year of the observation period (current costs) and the evaluation period (Future costs) as well as the log10 fold-change between them (**b**). Shown in (**c**, **d**) are the precision recall curves for predicting increasing and decreasing costs

Next, we analysed the ability of identifying patients with changing costs (Fig. 5a, b). In this analysis, we did not consider the model that used the last years costs as prediction for the future costs as costs are predicted to stay constant for this model. The results of the analysis in predicting patients with increasing/decreasing costs are shown in Fig. 5c, d, respectively. We found that overall, prediction of decreasing costs was easier than increasing costs. Furthermore, we found that for both direction of the cost change the neural network outperformed the ridge regression. For increasing costs the neural network had an auPRC of 0.08 while the ridge regression only had an auPRC of 0.04. For decreasing costs the neural network at an auPRC of 0.24 while the ridge regression had an auPRC of 0.21. A similar picture also emerged for the area under the ROC curve where the neural network had an auROC of 0.93 and 0.90 for decreasing and increasing costs, respectively. Here, the ridge regression had an auROC of 0.93 and 0.86 for decreasing and increasing costs, respectively. For both measures the Ridge regression and the neural network were substantially better than the baseline methods that did not model the costs.

**Fig. 6:**
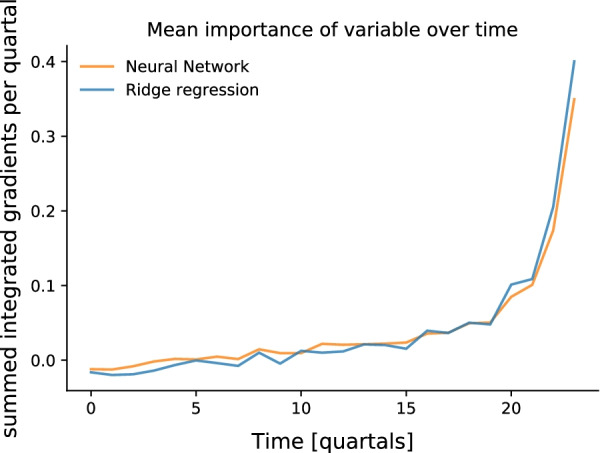
Importance of features per quarter: Shown is the summed normalized integrated gradient (Importance) per year for the ridge regression and the neural network

Finally, we studied via integrated gradients, on which features of the data the neural network based its prediction and how this differed from the features used by the ridge regression. We first determined the importance of features from different quarters in the observation period by summing the integrated gradients of all feature in a quarter. We found that both methods have a similar temporal distribution of the importance and that for prediction the most recent features were the most important (See Fig. 6).

To evaluate whether the features showed a qualitative difference between the neural network and the regression, we identified the features with the highest (associated with higher cost) integrated gradient in set of patients that have an 100-fold increase in costs. To this end, we summed the integrated gradients of each code over all quarters in the observation period. The top-20 codes are shown in Table 3 for the neural network and for the ridge regression in Table 4. We found that the neural network relied more on ICD-10-GM diagnosis and ATC medication code than the ridge regression (7 of 20 vs. 3 of 20).

**Table 3:** Shown are the top-20 codes with the highest feature importance as determined by the integrated gradients (IG) for the neural network, a high-level description of the codes as well as the corresponding IG

Code	Short description	IG
Sex Male	Male patient	0.017
GOP key 01321	Treatment by non health insurance-accredited physicians	0.014
GOP key 03004	Practitioner care of patients with age between 55 and 75 years	0.014
GOP key 03220	Treatment of a patient with at least one life-changing chronic disease	0.012
ICD-10-GM key F102	Alcohol dependence	0.011
Sex Female	Female patient	0.011
GOP key 03221	Treatment of a patient with at least one life-changing chronic disease	0.010
GOP key 03005	Practitioner care of patients older than 75 years	0.010
ICD-10-GM key F171	Mental and behavioural disorders due to use of tobacco	0.009
ICD-10-GM key M171	Unilateral primary osteoarthritis of knee	0.009
ATC code B01AC04	Clopidogrel (antithrombotic agent)	0.009
GOP key 03004R	Practitioner care of patients with age between 55 and 75 years (regional billing amendment to GOP 03004)	0.009
GOP key 32001A	Profitability bonus for arranging and providing laboratory services (regional billing amendment to GOP 32001)	0.008
GOP key 01731	Early detection of cancer in men	0.008
ICD-10-GM key M4809	Spinal stenosis	0.008
ICD-10-GM key G359	Multiple sclerosis	0.008
OPS key 80103	Application of drugs and electrolyte solutions via the vascular system in newborns	0.007
ATC code N06AX16	Venlafaxine (antidepressants)	0.007
GOP key 32001B	Profitability bonus for arranging and providing laboratory services (regional billing amendment to GOP 32001)	0.007
GOP key 07220	Surgical primary care	0.007

**Table 4:** Shown are the top-20 codes with the highest feature importance as determined by the integrated gradients (IG) for the ridge regression, a high-level description of the codes as well as the corresponding IG

Code	Short description	IG
GOP key 03220	Treatment of a patient with at least one life-changing chronic disease	0.020
GOP key 03221	Treatment of a patient with at least one life-changing chronic disease (intensified consultation of physician)	0.017
Sex Female	Female patient	0.015
GOP key 03004R	Practitioner care of patients with age between 55 and 75 years	0.014
GOP key 03004	Practitioner care of patients with age between 55 and 75 years	0.013
GOP key 03040	Family medical care	0.013
GOP key 03230	Problem-oriented medical consultation, which is necessary due to the nature and severity of the illness	0.010
ATC code V04CA02	Tests for diabetes (Glucose)	0.010
GOP key 03111R	Practitioner care of patients with age between 5 and 59 years	0.007
ATC code M01AE01	Ibuprofen (antiinflammatory and antirheumatic)	0.007
ICD-10-GM key F171	Mental and behavioural disorders due to use of tobacco	0.007
Hospital type	Hospitals of maximum care	0.006
GOP key 32001J	Profitability bonus for arranging and providing laboratory services	0.006
GOP key 03040G	General practitioner medical care	0.006
GOP key 32001A	Profitability bonus for arranging and providing laboratory services (regional billing amendment to GOP 32001)	0.006
FG key 53	Physician group: Neurology	0.005
GOP key 32094	Quantitative determination of glycated hemoglobins	0.005
GOP key 03212	Chronic disease	0.005
FG key 11	Physician group: Trauma Surgery	0.005

## Discussion

Accurate prediction of future health care cost provides the basis to optimally manage healthcare costs. Furthermore, identification of patients whose cost will change allows optimization of interventions given a limited budget in order to improve population health. To achieve this it is important to have accurate predictions of the future health costs. In this work we presented a deep learning based approach to predict future costs. Our approach can leverage the full complexity of the patient records and does not require prior feature selection.

We showed that our approach can outperform standard approaches, including the Morbi-RSA for all measured performance metrics (See Table 2). We suggest that the performance gain is due to two reasons. First, our approach learns important features from the data and does not require manual feature selection. It has been shown that learnt features allow better predictions in computer vision and speech processing given enough training data [19]). The value of learning predictive features from the data is suggested by the better (state-of-the-art) performance of our implementation of ridge regression compared to the existing implementation of Morbi-RSA that is only based on 80 diseases. Second, our deep learning approach allows modelling of complex interactions between all variables which is not possible for ridge regression. This enables better modelling of medical phenotypes such as interactions between age, sex and diagnosis. This is supported by the identified terms that are associated with increasing costs between ridge regression and the deep neural network, where the ridge regression uses mainly the GOP codes and the deep network puts a higher emphasis on medical diagnoses and prescribed drugs. It also worth noting that in contrast to the Morbi-RSA, which is mainly based on ICD-10-GM codes, both the ridge regression and the neural network rely on GOP codes.

Since we placed no strong assumption on the phenotype that we modelled, we believe that the neural network may also easily adapted to predict other medical phenotypes.

However, we also acknowledge that further research is necessary to better understand the merits and limits of deep learning in identifying medical phenotypes from insurance claims. This includes the optimal architecture of the networks but also strategies to interpret deep networks, to provide uncertainty estimates for the models and model distribution shifts caused by changes in billing regulations and treatment guidelines.

## Conclusion

Overall, we have shown that neural networks compare favorably to several baseline methods and that tools such as integrated gradients can be used to explain predictions. We therefore believe, that neural networks are a valuable addition to the toolkit that exist for working with population-size patient records. We acknowledge, however, that further research is needed to better understand the challenges, advantages and disadvantages of using neural networks for modeling other outcomes and patient trajectories from high-dimensional electronic patient records.

## Supplementary Information

**Additional file 1**. Contains the python code for the neural network.

## Data Availability

The datasets used in the current study are not publicly available due to privacy and security concerns. Code for the neural network can be found in the supplemental material.

## Abbreviations

ATC: Anatomical Therapeutic Chemical
auPRC: Area under the precision-recall curve
CPM: Cumming’s Prediction Measure
DRG: Diagnosis Related Group
OPS: German procedure classification
IG: Integrated Gradients
ICD-10-GM: International Statistical Classification Of Diseases And Related Health Problems, 10th revision, German Modification
InGef: Institute for Applied Health Research Berlin
MAPE: Mean absolute prediction error
GOP: Schedule of fees for physician outpatient services
