# Supplementary material for: Deep learning for prediction of population health costs
Source: BMC Med Inform Decis Mak. 2022 Feb 3;22:32. doi: 10.1186/s12911-021-01743-z (PMC8812208; doi:10.1186/s12911-021-01743-z)
Supplement: Supplementary file 1 — Additional file 1. Contains the python code for the neural network. [file 12911_2021_1743_MOESM1_ESM.pdf]

1 Supplement

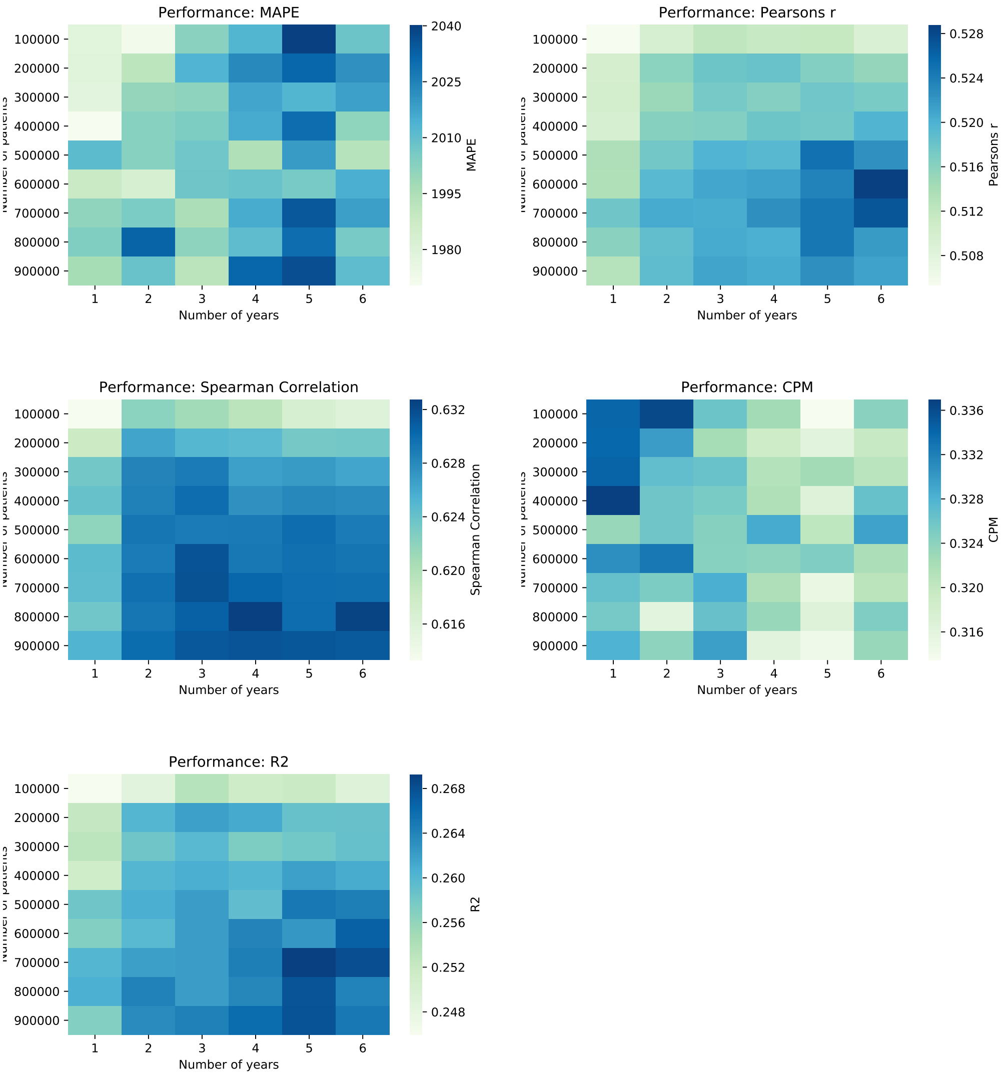

Figure 1: Dependence of performance on patient number and observation time: Shown is the performance (Pearson's correlation (r), Spearman's correlation (ρ), mean absolute prediction error (MAPE), R squared (r^2) and Cumming' s Prediction Measure (CPM) of the neural network depending on the patient number and the length of the observation period in years.

1.1 Python code for deep learning model

```python
import keras

from keras.layers import Input, Dense, Flatten
from keras.models import Model, Sequential
from keras import optimizers

model = Sequential()
inpt = Input(shape=(X_train.shape[1], X_train.shape[2]))
x_f = Flatten()(inpt)
x = Dense(50, input_shape=(X_train.shape[1] * X_train.shape[2],), acti
x = keras.layers.Dropout(0.25)(x)
x = Dense(50, activation='relu')(x)
x = keras.layers.Dropout(0.25)(x)
x = Dense(50, activation='relu')(x)
x = keras.layers.Dropout(0.25)(x)
x = Dense(50, activation='relu')(x)
x = keras.layers.Concatenate(axis=-1)([x, x_f])
outs = Dense(y_train.shape[1], activation='linear')(x)

model = Model(inputs=inpt, outputs=outs)

Adam = optimizers.Adam(clipnorm=1., amsgrad=True)

model.compile(loss='mean_squared_error',
        optimizer=Adam,
        metrics=['mse', 'mae'])
```
